# Navigating beyond the surface - prognostic significance of KRAS, NRAS, BRAF, PIK3CA, and TP53 mutations examined by exons

**DOI:** 10.3389/fonc.2025.1557609

**Published:** 2025-06-12

**Authors:** Vlad Adrian Afrăsânie, Mihai Vasile Marinca, Bogdan Gafton, Alexandra Rusu, Eliza Maria Froicu, Daniel Sur, Cristian Virgil Lungulescu, Raluca Cezara Popa, Irina Afrăsânie, Anca Viorica Ivanov, Georgiana Emmanuela Gîlcă-Blanariu, Lucian Miron, Cristina Rusu, Teodora Alexa-Stratulat

**Affiliations:** ^1^ Department of Medical Oncology, Regional Institute of Oncology, Iasi, Romania; ^2^ Department of Medical Oncology-Radiation Therapy, Faculty of Medicine, “Grigore T. Popa” University of Medicine and Pharmacy, Iasi, Romania; ^3^ Department of Medical Oncology, “Iuliu Hatieganu” University of Medicine and Pharmacy, Cluj-Napoca, Romania; ^4^ Department of Medical Oncology, The Oncology Institute “Prof. Dr. Ion Chiricuţă”, Cluj-Napoca, Romania; ^5^ Department of Oncology, University of Medicine and Pharmacy of Craiova, Craiova, Romania; ^6^ Department of Internal Medicine, Faculty of Medicine, “Grigore T. Popa” University of Medicine and Pharmacy, Iasi, Romania; ^7^ Department of Pediatrics, Faculty of Medicine, “Grigore T. Popa” University of Medicine and Pharmacy, Iasi, Romania; ^8^ Department of Gastroenterology, University of Medicine and Pharmacy “Grigore T. Popa”, Iasi, Romania; ^9^ Department of Genetics, Faculty of Medicine, “Grigore T. Popa” University of Medicine and Pharmacy, Iasi, Romania

**Keywords:** metastatic colorectal cancer, predictive biomarkers, prognostic biomarkers, KRAS, NRAS, BRAF, PIK3CA, TP53

## Abstract

**Introduction:**

Metastatic colorectal cancer (mCRC) is a disease with various molecular profiles that exhibit different evolution patterns. Although most mCRC patients receive the same chemotherapy drugs in the first-line setting, treatment response is heterogeneous suggesting some tumors are inherently resistant to oxaliplatin/fluoropyrimidine regimen. Genomic-based markers may help identify these patients and guidetreatment decisions due to potential prognostic and predictive value.

**Methods:**

We performed a retrospective analysis on 77 patients diagnosed with mCRC treated with an oxaliplatin/fluoropyrimidine regimen in the Regional Institute of Oncology Iaşi between April 2017 and December 2019. We studied the impact of KRAS, NRAS, BRAF, PIK3CA and TP53 genes and their mutations in a treatment-naive population.

**Results:**

The median progression free survival (PFS) was 11 months (95% CI, 10.2-11.7 months) and the median overall survival (OS) was 23.6 months (16.3-30.8 months). Multivariate analysis of factors affecting PFS revealed that KRAS exon –3 mutation was associated with quicker progression while on oxaliplatin-based chemotherapy. A similar analysis indicated that the KRAS exon –3 mutation was also associated with decreased OS (p=0.03). The presence of the TP53 in exon 8 was associated with an increased OS (p=0.001).

**Discussion:**

The present analysis offers insights into the prognostic implications of genes and exon-distributed mutations within RAS, BRAF, PIK3CA, and TP53 in mCRC. Subsequent prospective investigations with a more extensive patient cohort are needed to clarify the influence of exon-distributed mutations on therapeutic decision-making and prognostic outcomes.

## 1 Introduction

Colorectal cancer (CRC) is the second most common solid tumor in women and the third in men worldwide, with high incidence and mortality rates in Romania (1, 2). Recent advancements in metastatic CRC (mCRC) treatment have led to a significant improvement in overall survival, doubling from 10 to over 20 months (3). Key factors include enhanced staging, surgical techniques, and the introduction of new agents like oxaliplatin, irinotecan, and capecitabine, along with targeted therapies (bevacizumab, cetuximab, panitumumab) and checkpoint inhibitors (pembrolizumab, nivolumab) (4). However, this progress has been accompanied by rising healthcare costs and increased toxicity, with some patients experiencing rapid disease progression. Optimizing treatment selection to minimize side effects and identifying novel prognostic tools remains crucial (5). Biomarkers, such as microsatellite instability, which predicts response to checkpoint inhibitors, and RAS mutations for targeted therapies, are vital for personalized treatment. Emerging data suggest that other genetic mutations, including those within the same gene but in different exons, may also have significant prognostic and predictive value (6).

– Carcinogenesis in CRC involves the dysregulation of tumor suppressor genes, repair genes, and the activation of oncogenes such as KRAS, NRAS, BRAF, PIK3CA, and TP53. The RAS/RAF/MEK signaling pathway, crucial for cell proliferation and survival, is frequently altered in tumors and plays a key role in carcinogenesis (7–10). KRAS, a proto-oncogene located on chromosome 12, encodes a GTPase involved in cell division, differentiation, and apoptosis through the RAS/MAPK pathway, activated by the epidermal growth factor receptor (EGFR) (9, 10). In CRC, mutations at codons 12, 13, or 61 activate KRAS, leading to continuous signaling to the nucleus. RAS mutations are common in metastatic CRC (mCRC), occurring in up to 50% of cases, with exon 2 mutations being the most frequent, followed by mutations in exon 3 and exon 4 (11).

The clinical relevance of *KRAS* mutations in colorectal cancer has been established through several pivotal studies. The predictive role of KRAS was confirmed in landmark trials that demonstrated its association with resistance to anti-EGFR monoclonal antibodies. The pivotal study by Karapetis et al. (12) showed that patients with *KRAS*-mutant tumors derive no benefit from cetuximab, a finding also highlighted by Amado et al. for panitumumab (13). These results led to the incorporation of KRAS testing into routine clinical practice to guide treatment selection. The CRYSTAL trial conducted by Van Cutsen et al. further reinforced this, showing improved outcomes from anti-EGFR therapy only in patients with KRAS wild-type tumors (14). In addition to its predictive value, KRAS mutation status has been shown to have prognostic significance. Large-scale analyses by De Roock et al. and Roth et al. demonstrated that KRAS mutations are independently associated with poorer overall and progression-free survival. Together, these studies have positioned *KRAS* as a critical biomarker in the management of colorectal cancer (15, 16). However, less is known about the relationship between different types of mutations occurring in the RAS genes and patient outcome.

The BRAF V600E mutation defines a distinct subgroup of metastatic colorectal cancers (mCRC), and can be found in 8–10% of cases; it is associated with poor prognosis. The PETACC-3 and MRC COIN trials demonstrated that BRAF-mutant tumors are linked to significantly shorter overall survival, independent of other factors (16, 17). Of note, early studies had already suggested limited benefit from anti-EGFR therapies in BRAF-mutant mCRC (18). These pivotal findings confirmed BRAF V600E as both a negative prognostic and a positive predictive biomarker, guiding treatment selection in clinical practice.

PI3K is an important kinase in the PI3K/AKT1/mTOR pathway. This is a signaling pathway of EGFR and plays a significant role in cell growth, proliferation, and survival in multiple solid tumors (19). PIK3CA mutation occurs in 15-20% of CRC and is an activating mutation located in exon 9 or 20 (20). A recent meta-analysis confirmed that mutations in exon 20 of PIK3CA may serve as a marker of resistance to anti-EGFR treatment, although their prognostic significance remains controversial. However, there is also evidence contradicting these results (21).

TP53 mutations can be identified in 50–60% of CRC cases, particularly in left-sided and rectal tumors. They are associated with the inactivation of p53, a key regulator of DNA repair, apoptosis, and cell cycle arrest (21–23). While biologically significant, the clinical relevance of TP53 mutations as prognostic or predictive biomarkers remains a matter of debate. Some studies link TP53 mutations to poorer disease-free and overall survival (24), while others report no consistent correlation (25). A recent meta-analysis confirmed the inconsistent prognostic impact of TP53, suggesting that gain-of-function mutations in hypermethylated tumors may worsen prognosis (26). Additionally, TP53 and KRAS mutations together have been associated with increased chemoresistance and recurrence risk post-resection (27). Despite emerging evidence of a potential predictive role in KRAS wild-type tumors, further prospective validation is needed to establish TP53’s clinical utility in treatment selection (28).

The identification of key genetic mutations that contribute to cancer progression and metastasis has had a significant impact on drug discovery research. As such, *KRAS G12C* has recently emerged as an actionable target in metastatic colorectal cancer (mCRC). The KRYSTAL-1 trial demonstrated that adagrasib combined with cetuximab achieved a 34% response rate in previously treated patients, leading to FDA approval in 2024 (29). Similarly, the CodeBreaK 300 trial showed sotorasib plus panitumumab significantly prolonged progression-free survival compared to standard therapy (30). Other KRAS mutations, such as *G12D*, are under investigation with promising early-phase data (31). *BRAF V600E* mutations, associated with poor prognosis, became actionable with the BEACON CRC trial, where the triplet regimen of encorafenib, binimetinib, and cetuximab improved survival (32). The 2024 BREAKWATER trial expanded this approach to first-line treatment (33). *ERBB2 (HER2) amplification* also represents a key target in RAS/BRAF wild-type tumors. The HERACLES, MOUNTAINEER, and DESTINY-CRC02 trials demonstrated efficacy of HER2-targeted agents including trastuzumab, lapatinib, and trastuzumab deruxtecan (34–36).

Available findings indicate that genetic mutations are closely related to prognosis, response to treatment and in treatment resistance. However, because a significant part of the tests have been reported as a binary result – i.e. gene mutation present or absent, some correlations might have been under/over reported. Newer techniques such as Next Generation Sequencing (NGS) offer more details regarding each mutation and allow for a more in-depth analysis of prognostic and predictive significance, identifying potential markers of primary treatment resistance (37).

We conducted a retrospective study of mCRC patients from a Romanian tertiary center who were tested using NGS panel for mutations in KRAS, NRAS, BRAF, PIK3CA, and TP53 genes. The main aim of the study was to evaluate the prognostic role of these mutations and their subtypes for progression-free survival (PFS) and overall survival (OS). A secondary endpoint of our analysis was to identify other clinical and histopathological parameters that could impact PFS and OS.

## 2 Materials and methods

### 2.1 Study design

We performed a retrospective analysis involving patients diagnosed with metastatic CRC who had a pathology-confirmed colon or rectum adenocarcinoma diagnosis and received an oxaliplatin based chemotherapy regimen in the first-line setting. All cases for which the attending physician requested RAS testing be performed by the Molecular Biology department of the Regional Institute of Oncology (RIO) Iaşi from April 2017 to December 2019 were assessed for eligibility.

### 2.2 Subjects and data collection

For inclusion in the study, participants needed to fulfill the following criteria: age 18 or above at the time of diagnosis, histopathology report confirming colorectal carcinoma, confirmed metastatic stage based on imaging and/or pathology results and treated with oxaliplatin-based chemotherapy in the first-line setting. Patients that had received chemotherapy in the adjuvant setting or any other type of chemotherapy for CRC were excluded. The research adhered to the principles of the Declaration of Helsinki and received approval from the Ethics Committee of the Regional Institute of Oncology, Iaşi. The minimum number of samples required for analysis was assessed based on available literature data reporting on the mutation frequency of several KRAS, BRAF and TP53 exons in mCRC patients. We estimated an approximate frequency of 5% for exon-specific mutations of interest (11). Determining the optimal sample size required obtaining a minimum volume to ensure adequate representativeness of the patient category. To achieve this prerequisite, we established a 95% confidence interval and used the following equation accordingly:


$$n\geq {\left(Z_{\left(1-\frac{\alpha }{2}\right)}\right)}^{2}\times \frac{p\left(1-p\right)}{d^{2}}$$

with Z = 1.96 for a 95% confidence interval and a “d” value corresponding to an estimation error of 5%. For an assumed maximum error of 5%, the minimum sample size was estimated to be 72 cases.

**Figure 1:**
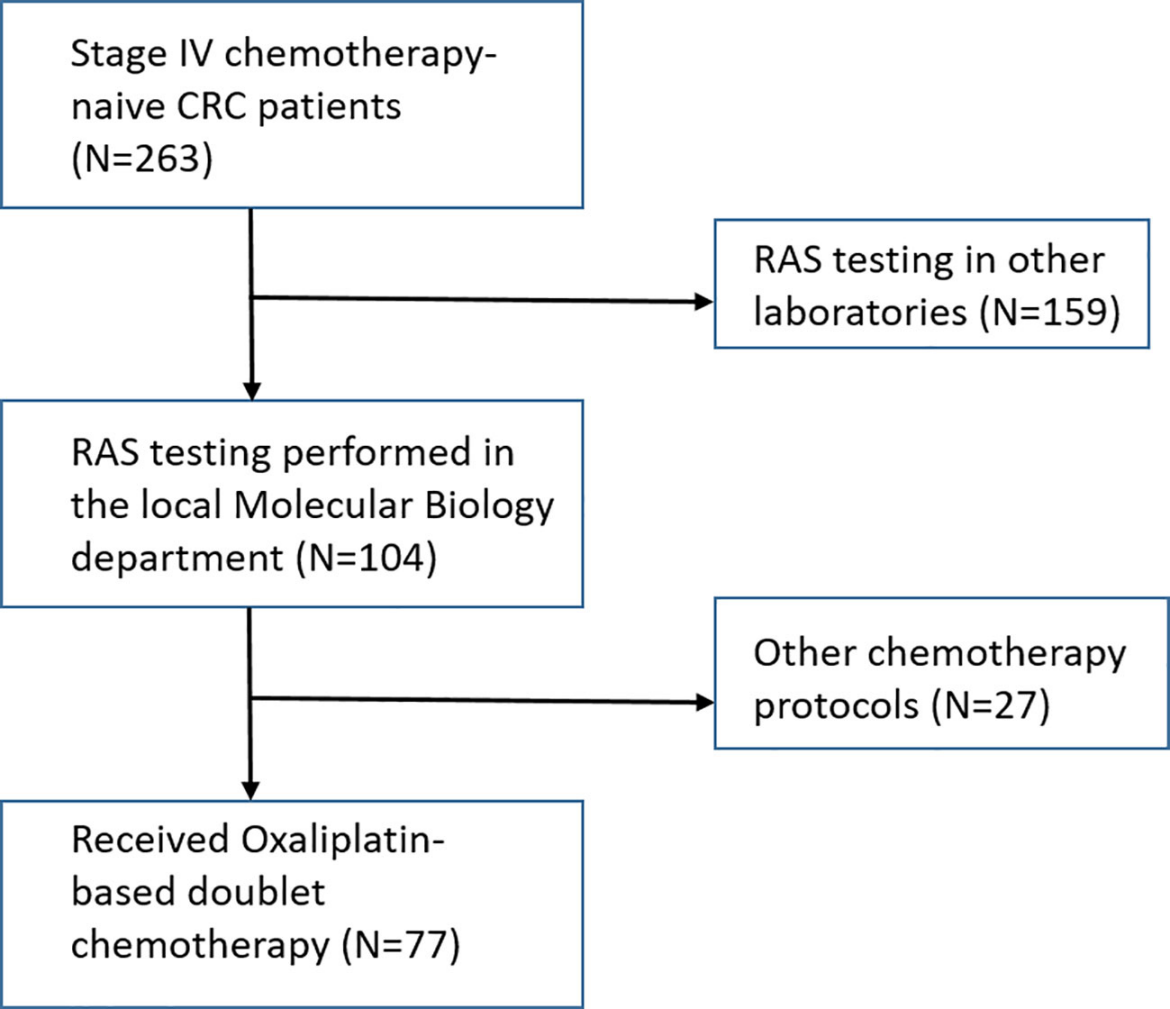
Patient selection flow-chart.

A total of 263 patients were diagnosed with stage IV CRC in the pre-specified time frame. Of those, 104 were tested for RAS mutation in our Molecular Biology department. 77 of the 104 patients received Oxaliplatin-based chemotherapy in the first-line setting. These individuals were tested for mutations in KRAS, NRAS, BRAF, PIK3CA, and TP53 genes from the time of diagnosis. Study flowchart can be seen in **Figure 1**.

### 2.3 Methods

The Department of Molecular Biology performed mutation screening on samples obtained from paraffin-embedded (FFPE) tumor tissue collected during colonoscopy or surgical resection. Five 10 μm thick sections of macrodissected FFPE were used to extract DNA. These sections had to contain at least 50% tumor epithelium, as determined by an experienced pathologist specialized in digestive tract tumors. Next-generation sequencing (NGS) was performed on all cases using the TruSight^®^ Tumor 15 kit for the Illumina platform, which allowed for a comprehensive analysis of 15 genes. Specifically, KRAS exons 2 (partial), 3 (partial), 4; NRAS exons 2 (partial), 3 (partial), 4; BRAF exon 15 (partial); PIK3CA exons 9 and 20; and TP53 exons were evaluated in every sample. It is important to underline that the TP53 gene was fully sequenced. All testing was conducted following the manufacturer’s instructions.

### 2.4 Statistical analysis

Various parameters were assessed within this database, including socio-demographic factors (age, gender), clinical characteristics (smoking status, ECOG performance status, primary tumor location, type of metastases), primary tumor surgery, and metastases removal), pathological (histotype, grading), molecular (KRAS, NRAS, BRAF, PIK3CA, TP53 mutations status and exon distribution), and survival parameters (PFS and OS).

Statistical analyses was performed by means of SPSS version 25.0 software (IBM Corporation, Armonk, NY, USA). Both overall survival (OS) and progression-free survival (PFS) estimates were determined using the Kaplan—Meier method. To discover potential predictive factors, a univariate analysis was used with the log-rank test, and a subsequent multivariate analysis was performed.

### 2.5 Aim of the study

The aim of this study was to assess the prognostic role of KRAS, NRAS, BRAF, PIK3CA, and TP53 mutations on PFS and OS in patients with mCRC who receive oxaliplatin based chemotherapy regimens. Additionally, we tried to evaluate the prognostic significance of certain clinical and histopathological parameters.

## 3 Results

### 3.1 Population and tumor characteristics

The average age at diagnosis was 63 years, with 45 (58.4%) of the patients being male. The majority of the patients (n=65, 84.4%) had an ECOG PS of 1. The primary tumor was most commonly located in the left colon (n=49, 63.6%). Metastases were most frequently found in the liver (n=47, 61%), followed by lung metastases (n=14, 18.2%). Among the patients, 50 (64.9%) underwent primary tumor surgery, and only 9 (11.7%) underwent surgical removal of the metastases. Additional data on clinical and pathological features of the studied group are reported in **Table 1**.

**Table 1:** Characteristics of the study population.

No. of Patients (%)	77 (100%)	
Median age (years)	63	
Sex	Male	45 (58.4)
	Female	32 (41.6)
Age	<65 years	40 (51.9)
	≥65 years	37 (48.1)
Smoking status	Non-smokers	72 (93.5)
	Smokers	5 (6.5)
ECOG	0	5 (6.5)
	1	65 (84.4)
	2	7 (9.1)
Primary tumor location	Left colon	49 (63.6)
	Right colon	28 (36.4)
Location of metastases	Liver	47 (61.)
	Peritoneal	11 (14.3)
	Lung	14 (18.2)
	Other sites	5 (6.5)
Histopathological type	Ulcerated	59 (76.6)
	Mucinous	15 (19.5)
	Signet ring cell	3 (3.9)
Grading	G1	6 (7.8)
	G2	65 (84.4)
	G3	6 (7.8)
Primary tumor surgery	No	27 (35.1)
	Yes	50 (64.9)
Metastases surgery	No	68 (88.3)
	Yes	9 (11.7)
First line chemotherapy regimen	FOLFOX	32 (41.6)
	CAPOX	45 (58.4)
First line biological treatment	Bevacizumab	41 (53.2)
	Cetuximab	12 (15.6)
	Panitumumab	4 (5.2)
	None	20 (26)

ECOG PS, Eastern Cooperative Oncology Group performance status.

Almost 50% of cases (44.2%) were found to have a KRAS mutation. NRAS mutations were less frequent, with six patients harboring changes in exon 2 or 3. Similarly low rates were recorded for BRAF mutations (2 patients) and PIK3CA mutations (5 patients). In contrast, almost 75% of patients harbored a TP53 mutation. The complete list of mutations identified can be seen in **Table 2**.

**Table 2:** Frequency of mutations in the study population.

Type of mutation	Exon location	Number of patients (%)
KRAS mutation	exon 2	30 (39)
	exon 3	5 (6.5)
	exon 4	4 (5.2)
NRAS mutation	exon 2	1 (1.3)
	exon 3	5 (6.5)
	exon 4	0 (0)
BRAF mutation	exon 15	2 (2.6)
PIK3CA	exon 9	2 (2.6)
	exon 20	3 (3.8)
TP53	exon 4	4 (5.2)
	exon 5	17 (22.1)
	exon 6	3 (3.9)
	exon 7	13 (16.9)
	exon 8	18 (23.3)
	exon 9	3 (3.8)
	exon 10	6 (7.8)
RAS wild type	No	42 (54.6)
	Yes	35 (45.4)
RAS BRAF wild type	No	42 (54.5)
	Yes	35 (45.5)
All wild type	No	34 (44.2)
	Yes	43 (55.8)

KRAS, Kirsten rat sarcoma viral oncogene homolog; NRAS, neuroblastoma ras viral oncogene homolog; BRAF, serine/threonine-protein kinase B-raf; PIK3CA, phosphatidylinositol-4,5-bisphosphate 3-kinase catalytic subunit alpha.

### 3.2 Survival outcomes

**Figure 2:**
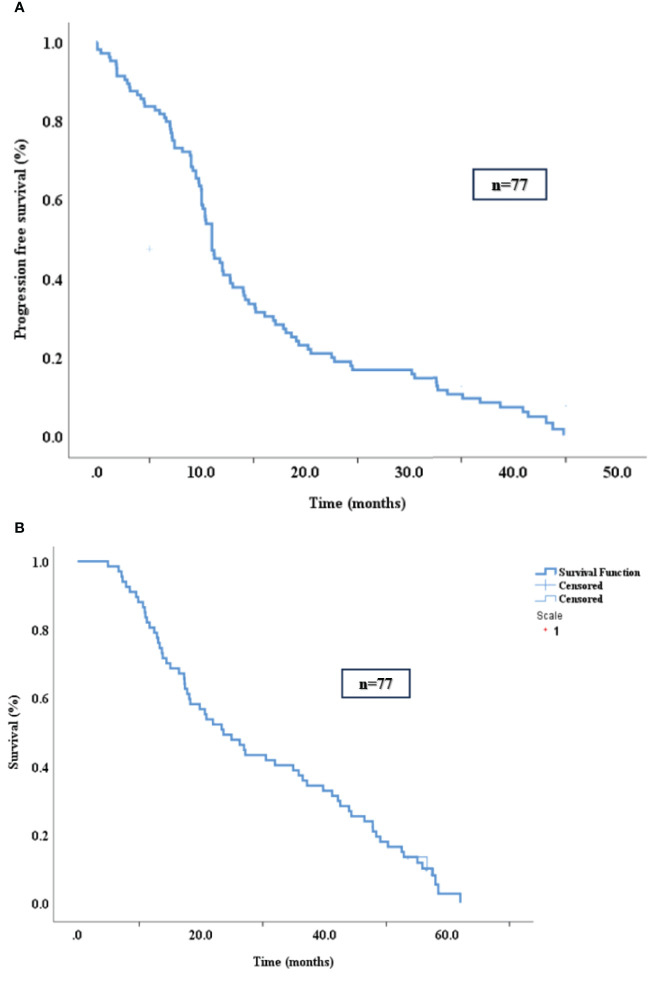
PFS **(A)** and OS **(B)** in the study population.

The median progression-free survival (PFS) in the studied cohort was 11 months (95% CI, 10.2-11.7 months) and the median overall survival (OS) was 23.6 months (16.3-30.8) as depicted in **Figure 2**.

### 3.3 Prognostic factors for PFS and OS

**Figure 3:**
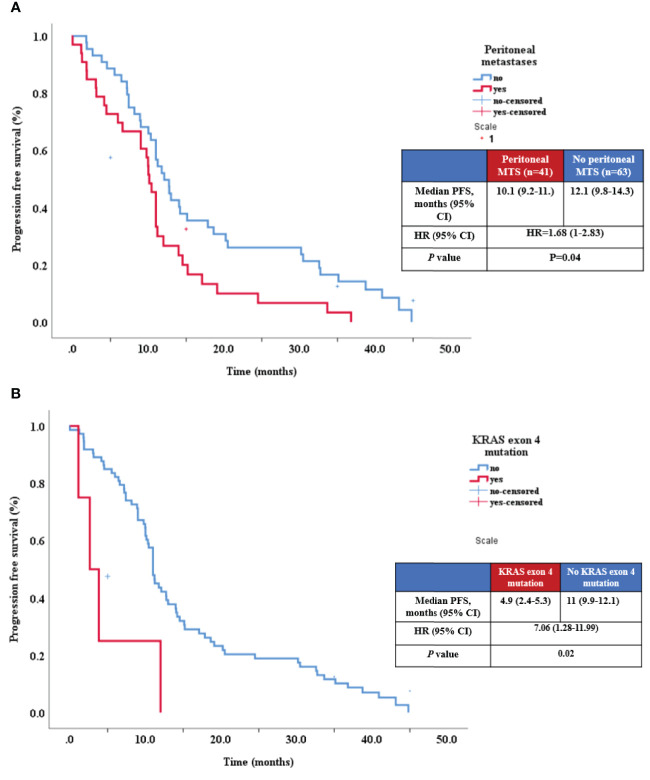
Relationship between PFS and presence of peritoneal metastases **(A)**, and KRAS exon 4 mutation **(B)**.

Univariate analysis indicated that PFS was shorter in patients with peritoneal metastases (p=0.01, HR=1.82, 95% CI 1.12-2.95). Several exon-specific mutations were associated with decreased PFS and the association was statistically significant: KRAS exon 3 mutation (p=0.002), KRAS exon 4 (p = 0.01) and TP53 exon 8 (p = 0.04) (**Table 3**; **Figure 3**).

**Table 3:** Univariate analysis of factors that influenced PFS.

Factor	HR	95% CI	*p* value
Sex	1.25	0.77-2.00	0.35
Age	0.92	0.57-1.46	0.72
Smoker status	1.09	0.43-2.72	0.85
ECOG PS	1.37	0.79-2.35	0.25
Primary tumor location	1.34	0.82-2.19	0.23
Liver metastases	0.77	0.48-1.24	0.29
**Peritoneal metastases**	**1.82**	**1.12-2.95**	**0.01**
Pulmonary metastases	1.33	0.67-2.61	0.4
Pathological subtype	0.84	0.55-1.3	0.44
Grading	1.38	0.68-2.81	0.36
Primary tumor surgery	0.82	0.53-1.27	0.38
First line chemotherapy regimen	1.11	0.69-1.79	0.64
First line biological treatment	0.83	0.68-1.01	0.06
Metastasectomy	1.23	0.6-2.5	0.55
**KRAS mutation**	**1.61**	**1-2.57**	**0.04**
KRAS exon 2 mutation	0.24	0.82-2.13	0.24
**KRAS exon 3 mutation**	**4.56**	**1.77-11.72**	**0.002**
**KRAS exon 4 mutation**	**3.76**	1.33-10.56	**0.01**
NRAS mutation	1.81	0.55-5.89	0.32
NRAS exon 2 mutation	1.91	0.26-14.03	0.52
NRAS exon 3 mutation	1.05	0.86-1.29	0.57
BRAF mutation	1.57	0.38-6.52	0.52
PIK3CA mutation	1.16	0.41-3.24	0.76
PIK3CA exon 9 mutation	1.23	0.29-5.1	0.77
PIK3CA exon 20 mutation	0.71	0.22-2.28	0.57
TP53 mutation	1.06	0.63-1.78	0.82
TP53 exon 4 mutation	1.47	0.53-4.1	0.45
TP53 exon 5 mutation	1.61	0.91-2.82	0.09
TP53 exon 6 mutation	0.58	0.14-2.41	0.46
TP53 exon 7 mutation	0.66	0.35-1.25	0.2
**TP53 exon 8 mutation**	**0.56**	**0.32-0.99**	**0.04**
TP53 exon 9 mutation	2.27	0.7-7.36	0.16
TP53 exon 10 mutation	1.35	0.58-3.14	0.47
**RAS wild type**	**0.57**	**0.35-0.92**	**0.02**
**RAS BRAF wild type**	**0.55**	**0.34-0.88**	**0.01**
**ALL WT**	**1.66**	**1.03-2.67**	**0.03**

ECOG PS, Eastern Cooperative Oncology Group performance status; KRAS, Kirsten rat sarcoma viral oncogene homolog; NRAS, neuroblastoma ras viral oncogene homolog; BRAF, ser-ine/threonine-protein kinase B-raf; PIK3CA, phosphatidylinositol-4,5-bisphosphate 3-kinase cat-alytic subunit alpha.

Bold values represent instances where p<0.05 (statistical significance).

**Figure 4:**
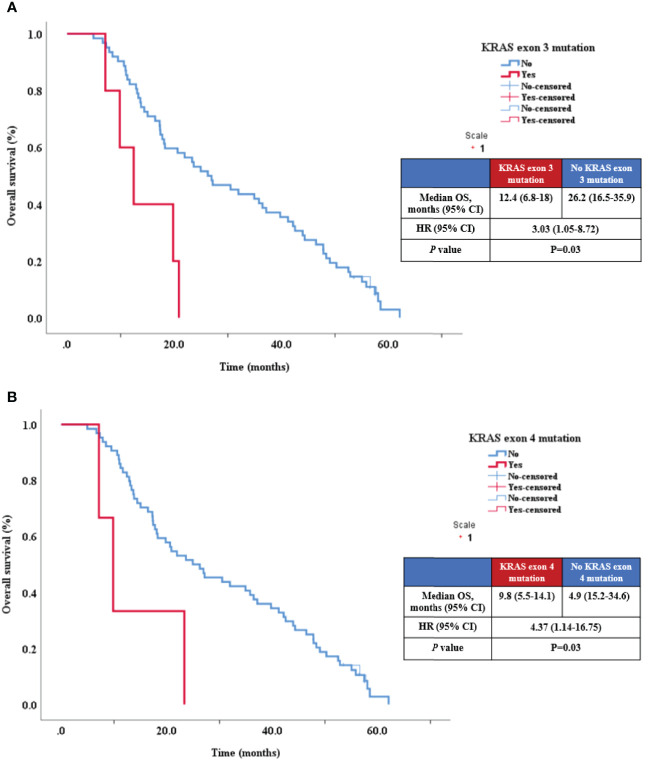
Relationship between OS KRAS exon 3 mutation **(A)** and KRAS exon 4 mutation **(B)**.

**Figure 5:**
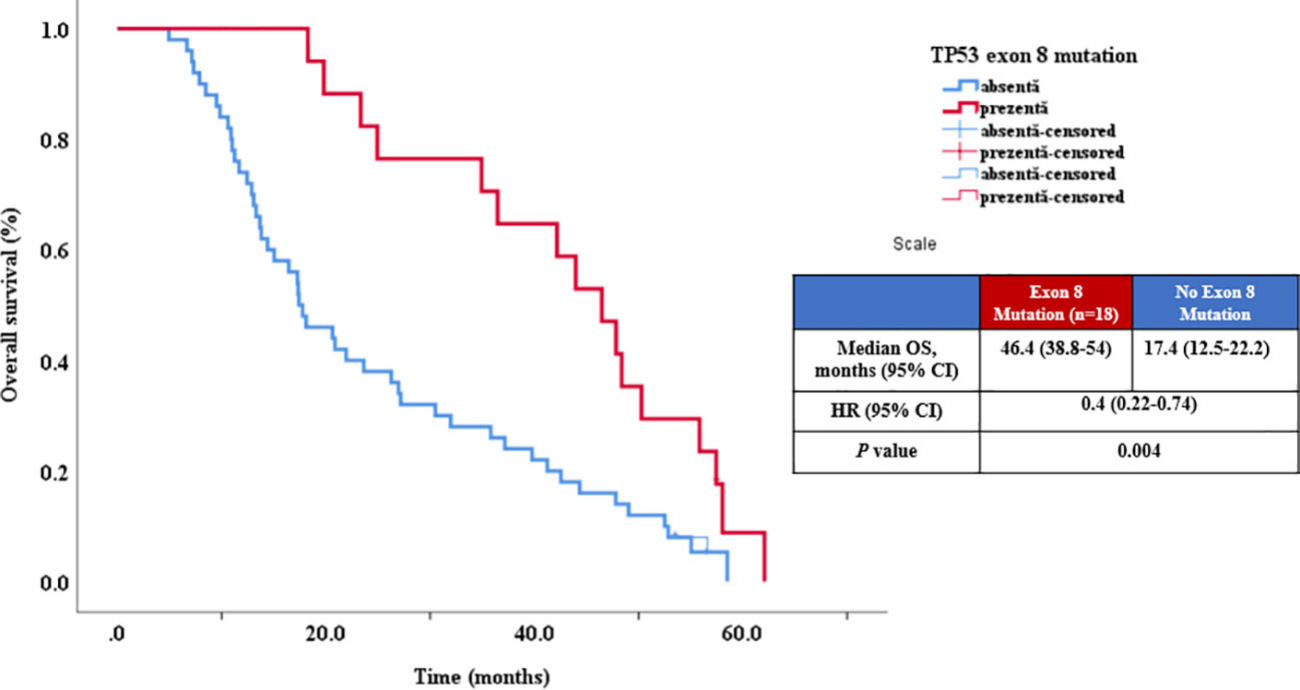
Relationship between OS and TP53 exon 8 mutation.

Similarly, univariate analysis for OS concluded that survival had a statistical tendency of being shorter in patients with peritoneal metastasis (p=0.07, HR=1.58, 95% CI 0.96-2.61) and also identified several mutations with potential OS impact: KRAS exon 3 (p = 0.01), (**Figure 4**), KRAS exon 4 (p = 0.02), (**Figure 4**) and TP53 exon 8 (p = 0.004) (**Table 4**, **Figure 5**).

**Table 4:** Clinical factors, pathological factors and genetic biomarkers and their impact on OS.

Factor	Univariate analysis		
	HR	95% CI	*p* value
Sex	0.89	0.52-1.5	0.67
Age	1.01	0.61-1.66	0.96
Smoker status	1.22	0.43-3.43	0.7
ECOG PS	1.37	0.75-2.48	0.29
Primary tumor location	0.85	0.5-1.45	0.55
Liver metastases	0.95	0.57-1.58	0.84
Peritoneal metastases	1.58	0.96-2.61	0.07
Pulmonary metastases	0.59	0.26-1.34	0.21
Pathological subtype	0.94	0.59-1.5	0.82
Grading	1.78	0.72-4.38	0.2
Primary tumor surgery	1.11	0.64-1.91	0.7
Metastasectomy	1.03	0.5-2.1	0.92
First line chemotherapy regimen	1.21	0.72-2.04	0.46
First line biological treatment	0.97	0.78-1.2	0.8
KRAS mutation	1.3	0.79-2.14	0.29
KRAS exon 2 mutation	1.09	0.66-1.8	0.73
**KRAS exon 3 mutation**	**3.6**	**1.37-9.43**	**0.01**
**KRAS exon 4 mutation**	**3.84**	**1.16-12.64**	**0.02**
NRAS mutation	1.15	0.28-4.77	0.84
NRAS exon 2 mutation	1.57	0.21-11.54	0.65
NRAS exon 3 mutation	2.81	1-7.88	0.04
BRAF mutation			
PIK3CA mutation	1.01	0.31-3.26	0.97
PIK3CA exon 9 mutation	2.9	0.68-12.31	0.14
PIK3CA exon 20 mutation	0.54	0.13-2.22	0.39
TP53 mutation	0.61	0.33-1.13	0.11
TP53 exon 4 mutation	1.84	0.65-5.21	0.24
TP53 exon 5 mutation	1.66	0.91-3.03	0.09
TP53 exon 6 mutation	1.21	0.29-5.02	0.78
TP53 exon 7 mutation	0.66	0.35-1.25	0.2
**TP53 exon 8 mutation**	**0.41**	**0.23-0.75**	**0.004**
TP53 exon 9 mutation	3.19	0.95-10.65	0.06
TP53 exon 10 mutation	1.31	0.56-3.07	0.52
RAS wild type	0.75	0.45-1.24	0.26
RAS BRAF wild type	0.75	0.45-1.24	0.26
ALL WT	1.41	0.85-2.34	0.17

ECOG PS, Eastern Cooperative Oncology Group performance status; KRAS, Kirsten rat sarcoma viral oncogene homolog; NRAS, neuroblastoma ras viral oncogene homolog; BRAF, ser-ine/threonine-protein kinase B-raf; PIK3CA, phosphatidylinositol-4,5-bisphosphate 3-kinase cat-alytic subunit alpha.

Bold values represent instances where p<0.05 (statistical significance).

For the multivariate analysis, we decided to include both statistically significant and clinically significant variables. Taking into account the fact that both exon 3 and exon 4 KRAS mutations were shown to have a statistically significant impact on both OS and PFS, but the two were mutually exclusive, we only chose KRAS exon 3 mutation for multivariate analysis due to a higher HR value when compared to KRAS exon 4 mutation. Our multivariate analysis also included TP53 exon 8 mutation (statistically significant for both PFS and OS in univariate analysis) and peritoneal metastasis (statistically significant for PFS and with a statistically significant trend for OS). In addition, we included several clinical variables that did not show a statistically significant impact on OS or PFS, but are well-known prognostic factors: ECOG performance status, tumor grading, metastasis surgery, and tumor histology. To be considered as having good predictive power, the model and its significance were evaluated. The probability of observing the real situation was assessed by applying the likelihood ratio test. This tests the difference -2LL (likelihood ratio) between the complete model with predictors and the initial one without predictors (null model) based on the Chi-square test (Omnibus Tests of Model Coefficients). The results indicated that the model can correctly evaluate a significant number (χ2 = 24.538; p=0.006) of cases (-2LL=482.034).

Multivariate analysis indicated that KRAS exon 3 mutation was significantly associated with a decreased PFS. While TP53 exon 8 and peritoneal metastasis exhibited a statistical tendency, p values did not reach.05 (**Table 5**). In the OS analysis, KRAS exon 3 mutation was again identified as a negative prognostic factor (p = 0.033, HR = 3.14). However, TP53 exon 8 mutation was associated with good prognosis (**Table 6**).

**Table 5:** Multivariate analysis of factors that impact PFS.

Variables in the equation						
Factor	B	SE	Sig.	Exp (B) HR	95.0% CI for Exp (B)	
					Lower	Upper
ECOG (0 Ref.)						
ECOG (1)	.527	.657	.422	1.694	.467	6.141
ECOG (2)	.460	.764	.547	1.585	.354	7.089
Peritoneal metastasis (0= NO Ref.)	.490	.275	.074	1.632	.953	2.797
Metastasectomy (1= YES Ref.)	-.310	.394	.432	.733	.338	1.589
Grading (1=G1 Ref.)						
Grading 2=G2	-.071	.494	.886	.931	.354	2.453
Grading 3=G3	.396	.643	.538	1.485	.421	5.234
Histology 1=ADK Ref.						
Histology (2=Mucinos)	-.042	.334	.901	.959	.499	1.844
Histology (3=Signet ring)	-1.005	.799	.209	.366	.076	1.753
KRAS exon 3 mutation (0 =NO Ref.)	1.118	.526	.034	3.060	1.091	8.579
TP53 exon 8 mutation (1 = YES Ref.)	.549	.314	.080	1.732	.936	3.202

Cox Regression. Method: Enter.

Omnibus Tests: -2 Log Likelihood=482.034; Chi-square=24.538; p=0.006.

**Table 6:** Multivariate analysis of factors that impact OS.

Variables in the equation						
Factor	B	SE	Sig.	Exp (B) HR	95.0% CI for Exp (B)	
					Lower	Upper
ECOG (0 Ref.)			.270			
ECOG (1)	.935	.601	.120	2.548	.785	8.276
ECOG (2)	1.096	.745	.141	2.993	.695	12.892
Peritoneal metastasis (0= NO Ref.)	.088	.303	.771	1.092	.603	1.977
>Metastasectomy (1= YES Ref.)	.205	.398	.607	1.227	.563	2.676
Grading (1=G1 Ref.)			.406			
Grading 2=G2	.337	.796	.672	1.400	.294	6.660
Grading 3=G3	1.070	1.001	.285	2.915	.410	20.735
Histology 1=ADK Ref.			.500			
Histology (2=Mucinos)	-.432	.380	.255	.649	.308	1.366
Histology (3=Signet ring)	.094	.656	.886	1.099	.304	3.974
KRAS exon 3 mutation (0 =NO Ref.)	1.145	.538	.033	3.144	1.096	9.018
TP53 exon 8 mutation (1 = YES Ref.)	1.112	.341	.001	3.040	1.557	5.935

Cox Regression. Method: Enter.

Omnibus Tests: -2 Log Likelihood=402.534; Chi-square=25.263; p=0.005.

## 4 Discussions

In the past few years, prognostic and predictive factors in mCRC have been thoroughly investigated. Some of these factors are now widely accepted and used in daily practice, such as stage, metastatic site or resection of the primary tumor, while others are still being assessed (38). In terms of genomic markers, any RAS or BRAFV600E mutation is associated with primary resistance to cetuximab and panitumumab. Resistance to first-line oxaliplatin-based chemotherapy is more difficult to predict. A recent article suggested that a specific gene signature constructed based on four oxaliplatin resistance-related genes is highly prognostic for the survival of colon cancer patients. Using two different large gene sets, the authors obtained gene expression profiles and noted that there were several differently expressed genes in the oxaliplatin-resistant compared to the oxaliplatin-sensitive colon cancer cells. Subsequently, these genes were screened and key oxaliplatin-resistance genes were selected that were then used to build a risk model for colon cancer patients. However, these genes were characterized via extensive sequencing and while NGS analysis has offered significant insight into prognosis and treatment resistance for various tumor types, both cost and availability are still an issue in many parts of the world. Clinical-grade genomic biomarkers represent a practical alternative that can guide treatment decisions while concurrently offering prognostic and predictive information (39).

Literature data reporting on associations between KRAS mutations and survival are abundant, although not entirely in concordance (40). In recent phase III clinical trials assessing the efficacy of anti-EGFR agents, it has been observed that patients with mCRC with exon 3 and 4 KRAS mutations did not respond as well to the treatment. However, these distinct subgroups have been together analyzed as a single category due to the small number of patients and have not been examined separately (6).

In our study we analyzed the predictive and prognostic role of different exons. While exon 3 mutations make up only 1% of colorectal cancers, their prognostic significance has been explored in a limited number of studies. In our investigation, the presence of a KRAS exon 3 mutation emerged as an independent prognostic factor associated with decreased OS. Our results are similar to those obtained by Lavacchi et al. who conducted an observational study on the prognostic and predictive value of KRAS mutations in mCRC. The authors concluded that individuals with mutations on exon 3 codon 61 exhibited the lowest median OS among the entire analyzed population (median OS 4.0 months, 95% CI: 3.5–NR). The same study identified a notable discrepancy in median PFS between exons 2 and 4 compared to exon 3 (exon 2: 9.7 months, exon 4: 10.5 months, exon 3: 4.3 months). On the other hand, some studies have indicated that KRAS exon 3 mutations may present a less aggressive biological behavior than exon 2 mutations, as they were associated with a lower TNM stage and a less invasive tumor (6, 41–43).

In the present study, KRAS exon 4 mutations were associated with a reduced PFS. The presence of KRAS exon 4 mutations also impacted the prognosis, leading to a lower OS. However, our data is distinct from results obtained by other retrospective observational studies that have concluded that KRAS mutations in exon 4 were associated with a positive outcome in patients with mCRC. In a study by Frankel et al., encompassing 165 patients with mCRC and resected hepatic metastases, the cohort of patients harboring mutations in exon 4 demonstrated a markedly superior 5-year disease-specific survival rate at 83%, in contrast to those with mutations in exon 2 (35%) and those without any K/NRAS mutation (54%) (P < 0.05) (41). Similarly, in the study by Lavacchi et al., encompassing 183 KRAS-mutated patients with stage IV disease, the authors observed that patients with KRAS exon 4 mutations had a median PFS of 10.4 months, in comparison to exon 2 (9.7 months) and exon 3 (4.3 months) mutations, with a statistically significant p-value of 0.027. A superior median OS was noted in patients with KRAS mutations in exon 4, with an median OS of 43.6 months (95% CI, 28.2-NR) compared to other exons, which had an OS of 20.6 months (95% CI, 5-24.7, HR=0.45; 95% CI, 0.21-0.99; P=0.04) (6). A possible explanation for this discrepancy is that these other patient cohorts included all types of first-line chemotherapy, whereas our analysis was specifically focused on patients receiving a combination of oxaliplatin and fluoropyrimidines. A recent analysis by Iris van ‘t Erve et al. regarding the role of exon 4 mutations suggested that the A146 mutation appears to be associated with a more unfavorable prognosis than G12 isoforms. Differences between various isoforms could potentially influence RAS signaling differently by modulating the balance between active and inactive forms concerning GAP (G12 and Q61) or GEF, or both (44).

The present study has showed the prognostic heterogeneity within a large cohort of KRAS mutations, attributable to their distinct ability to exert either deleterious or beneficial influences on overall survival, highlighting the unmet need for dedicated attention to this subgroups and to stop consider only the mutational status of the genes.

When the TP53 gene was examined as a predictive and prognostic factor, it was observed that exon 8 was associated with increased OS, while exon 9 was associated with decreased OS. There are few studies in the research oncology community which analyzed exclusively the role of TP53 mutations by exons in mCRC. Regarding the data from literature, in a study involving 161 patients with mCRC, the TP53 mutation status was evaluated as a prognostic factor in patients with left-sided colon tumors treated with chemotherapy and anti-EGFR therapies. mCRC patients with TP53 mutation exhibited a negative prognostic, with a significantly reduced OS with approximately 20 months than patients who did not receive anti-EGFR (45). In an Asian study encompassing patients with colorectal cancer (CRC) stages I-IV, it was observed that mutations in exon 5 and 7 of the TP53 gene served as prognostic factors. This finding suggests that these mutations may have significance in the prognosis of CRC. However, the subgroup of patients with stage IV was not analyzed separately (46). In a meta-analysis investigating the prognostic role of the TP53 gene in mCRC, nine retrospective studies were included, and they did not support the prognostic role for the TP53 gene. It is important to note that the selected studies exhibited significant heterogeneity in terms of genetic sequencing techniques. Additionally, only one study addressed the prognostic effect of gain or loss-of-function mutations, which could potentially have distinct prognostic implications. Also, the meta-analysis did not offer any details about the prognostic role of exons (28).

Our study provides an overview of RAS, BRAF, PIK3CA, and TP53 mutations as prognostic factors, raising specific observations and supplementing the limited literature data regarding the localization on exons. Nevertheless, it is imperative to acknowledge that our study has certain limitations, primarily pertaining to methodology. We lacked a sizable, highly selected, and well-balanced study population, potentially impacting the precision of our results.

Future research are warranted with prospective studies on large patient cohorts with improved methodologies to elucidate the role of this gene and the prognostic and predictive role of every exon.

## 5 Conclusions

This retrospective, single-center analysis provides insights into the predictive and prognostic roles of genes and exon-distributed mutations in RAS, BRAF, PIK3CA, and TP53 in mCRC. Prospective studies with a larger patient cohort are warranted to elucidate the impact of exon-distributed mutations on therapeutic decision-making and prognostic outcomes. We consider that this manuscript may offer preliminary data and clues for future investigations in this research area.

## Data Availability

The raw data supporting the conclusions of this article will be made available by the authors, without undue reservation.
